# Efficacy of a digitally designed anatomical repositioning splint for adolescent temporomandibular joint anterior disc displacement with reduction: a randomized controlled trial

**DOI:** 10.1186/s12903-026-08753-1

**Published:** 2026-06-05

**Authors:** Wenfang Wang, Meijuan Wang, Jun’an Wu, Yangwei Xu, Ziyu Huang, Zhenquan Xing, Shuang Wang

**Affiliations:** 1 Key Laboratory of Shaanxi Province for Craniofacial Precision Medicine Research, College of Stomatology, Xi’an Jiaotong University, 98 Xiwu road, Xi’an, 710004 China; 2 Anesthesiology Department, Peking University Shenzhen Hospital Xinhua Campus (Shenzhen Xinhua Hospital), Shenzhen, 518036 China; 3 Clinical Research Center of Shaanxi Province for Dental and Maxillofacial Diseases, College of Stomatology, Xi’an Jiaotong University, Xi’an, 710004 China; 4 Anesthesiology Department, Guangdong Provincial People’s Hospital, Guangzhou, 510080 China

**Keywords:** Anterior Disc Displacement with Reduction, Anterior repositioning splint group, Digital design and fabrication

## Abstract

**Background:**

Anterior disc displacement with reduction (ADDwR) is a common temporomandibular joint disorder in adolescents. Conventional anterior repositioning splints (ARS) often lack anatomical adaptation, which may limit their therapeutic precision and patient comfort. This study aimed to compare the efficacy of a digitally designed anatomical repositioning splint (D-ARS) with that of a conventional ARS in adolescent ADDwR patients.

**Methods:**

In this randomized controlled trial, 82 adolescents (aged 12–16 years) with ADDwR were randomly allocated to receive either a D-ARS (*n* = 41) or an ARS (*n* = 41). At the 6‑month follow‑up, clinical outcomes were evaluated through three modalities: disc recapture was assessed by magnetic resonance imaging (MRI), condylar volume changes were measured using cone‑beam computed tomography (CBCT), and patient‑reported wearing comfort was quantified with the Oral Impacts on Daily Performance (OIDP) questionnaire. Statistical analyses included chi-square tests, t-tests, and non-parametric tests as appropriate.

**Results:**

The D-ARS group achieved a significantly higher disc recapture rate than the ARS group (94.44% vs. 79.63%, *p* = 0.022). Both groups showed significant increases in condylar volume [D-ARS: +202.17 mm³ (95% CI: 177.72–226.63), ARS: +208.9 mm³ (95% CI: 178.8–241.25), both *p* < 0.001], with no intergroup difference (*p* = 0.5615). Patient-reported comfort was superior in the D-ARS group, with lower OIDP scores (14.43% vs. 35.63%, *p* < 0.001).

**Conclusions:**

At the 6-month follow-up, the D-ARS group exhibited significantly better disc recapture success and patient-reported wearing comfort than the ARS group, while no significant difference was observed between the two groups in terms of condylar growth promotion.

**Trial registration:**

Chinese Clinical Trial Registry (ChiCTR2400080947, registered on February 19, 2024). Available from: http://www.chictr.org.cn/.

**Supplementary Information:**

The online version contains supplementary material available at 10.1186/s12903-026-08753-1.

## Introduction

Temporomandibular disorders (TMD), as a common condition affecting the orofacial region, are clinically characterized by joint pain, functional impairment, and structural abnormalities, which can significantly reduce patients’ quality of life in severe cases. Epidemiological data indicate that the prevalence of TMD in adult populations ranges from 25% to 50%, establishing it as one of the most prevalent oral health issues following dental caries, periodontal disease, and malocclusion, and is associated with considerable psychological stress and socioeconomic burden [1]. In recent years, studies have shown that the prevalence of TMD in children and adolescents is similarly noteworthy, with reported rates reaching 20%–60% [2]. Anterior disc displacement, a common pathological manifestation, may interfere with normal condylar growth and development, potentially leading to dentofacial abnormalities [3]. Thus, early identification and intervention of TMD in adolescents are of critical importance for preventing long-term functional impairment and morphological deformities [4].

Currently, the management of anterior disc displacement with Reduction (ADDwR) primarily relies on the use of anterior repositioning splints (ARS). By adjusting the mandibular position to achieve disc recapture, ARS has been widely recognized as a non-invasive and effective treatment modality [5]. However, conventional ARS presents several clinical limitations. Notably, the lack of well-defined occlusal contacts in the posterior region restricts masticatory function during wear. Additionally, the bulky anterior ramp often interferes with speech and compromises comfort, which may reduce patient adherence, increase the risk of disc recapture failure, and lead to higher dropout rates during treatment [6]. These shortcomings constrain further improvement in therapeutic outcomes, underscoring the need to explore more precise and patient-friendly treatment approaches.

The rapid advancement of digital technology has brought new opportunities for the diagnosis and treatment of oral and maxillofacial diseases. Digital design and fabrication technologies enable the precise reconstruction of occlusal relationships based on individual anatomical features, allowing for the personalized fabrication of occlusal splints, enhancing clinical efficiency, accuracy, and outcome predictability [7, 8]. By adopting a digital workflow, we modified the conventional ARS by eliminating the oversized anterior ramp and incorporating anatomical posterior cusp morphology. The splint was further customized according to the individual’s mandibular movement trajectory to ensure optimal fit with the patient’s unique oral anatomy. We refer to it as the “digitally designed anatomical repositioning splint (D-ARS)”. These modifications were intended to improve wearing comfort and adherence, thereby potentially increasing the efficacy of disc recapture and overall treatment experience.

The current study compared the clinical efficacy and wearing comfort of D-ARS and that of ARS in adolescent ADDwR patients. The null hypothesis postulates no difference in the clinical performance of D-ARS and ARS for adolescents with ADDwR.

## Methods

### Trial design

**Fig. 1:**
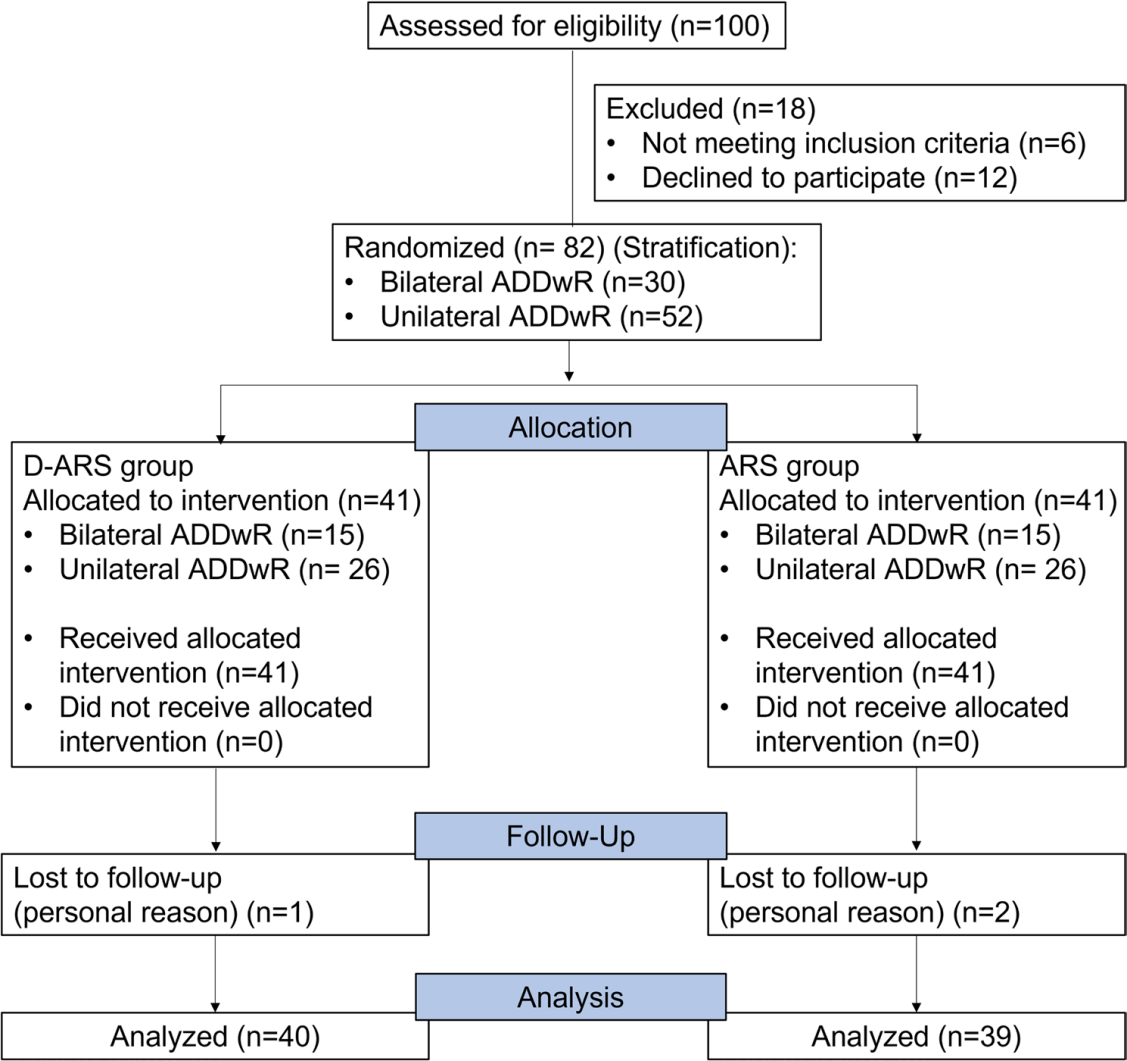
Study flow diagram

This was a parallel-group, superiority randomized controlled trial conducted in accordance with the Consolidated Standards of Reporting Trials (CONSORT) 2025 guidelines [9] (see Supplementary Material 4 for the completed CONSORT checklist). The primary objective was to determine whether the D-ARS is superior to the ARS in achieving disc recapture in adolescent patients with ADDwR. Sample size calculation was based on the anticipated difference in the primary outcome (disc recapture rate). This trial was registered with the Chinese Clinical Trial Registry (registration number: ChiCTR2400080947; registration date: February 19, 2024). The full trial protocol and statistical analysis plan are available at http://www.chictr.org.cn/. Consort flow chart, Fig. 1.

### Protocol amendments and Harms

No important changes were made to the trial protocol after its commencement. All outcomes and analyses reported in this manuscript were pre-specified in the registered protocol (ChiCTR2400080947) and conducted as originally planned.

Harms were defined as any adverse events or unintended effects related to the study interventions. At each follow-up visit (1 week, 1 month, 3 months, and 6 months), patients were systematically asked about any discomfort, pain, functional impairment, or other symptoms potentially associated with splint wear. All reported harms were documented and assessed for severity and relationship to the intervention by the treating clinician.

### Ethical clearance

This study protocol was reviewed and approved by the Medical Ethics Committee (Approval No. 2024-XJKQIEC-XJS-0009-001). The trial was conducted in full accordance with the ethical principles established in the Declaration of Helsinki (2013 revision). Prior to enrollment, written informed consent was obtained from the legal guardians of all participating adolescents, and age-appropriate assent was also secured from the participants themselves before initiating any study-related procedures. Patients were involved solely as study participants and did not take part in the design, conduct, or reporting of this trial.

### Sample size

The sample size was calculated based on the primary outcome (disc recapture rate). Assuming a success rate of 95% in the D-ARS group and 75% in the ARS group (Based on previous literature [10–12]), with a two-sided alpha of 0.05 and power (1-β) of 80%, a minimum of 37 patients per group was required (calculated using G-Power 3.1). Accounting for an estimated 10% loss to follow-up, we aimed to recruit 82 patients (41 per group).

### Patient eligibility

#### Inclusion criteria for both groups

1. Adolescents aged 12–16 years old.
2. Unilateral or bilateral ADDwR [confirmed by magnetic resonance imaging (MRI)].
3. Full permanent dentition (excluding third molars).
4. Good periodontal health with no clinically detectable tooth mobility.
5. No missing teeth (congenital or extracted).
6. Absence of active pulpitis or apical periodontitis.
7. Good general health with no history of maxillofacial trauma or systemic diseases affecting the TMJ, such as rheumatoid arthritis.
8. Scheduled for treatment with an anterior repositioning splint.

#### Exclusion criteria for both groups

1. Unilateral anterior disc displacement without reduction (confirmed by MRI)
2. Presence of severe psychiatric or psychological disorders (e.g., major depression, anxiety disorders) that may compromise treatment adherence or affect pain perception.
3. History of maxillofacial trauma or previous TMJ surgery.
4. Presence of systemic arthritis or connective tissue disease.
5. Current or previous orthodontic/orthognathic treatment.
6. Inability to comply with follow-up visits or splint wear protocol.

#### Recruitment

Participants were consecutively recruited from the Temporomandibular Joint Clinic of Hospital of Stomatology Xi’an Jiaotong University, Xi’an, China, between February 2024 and June 2025.

#### Intervention provider qualifications

All study procedures were performed by qualified orthodontists specialized in temporomandibular disorders. Digital design and fabrication of D-ARS were conducted by a trained dental technician with expertise in CAD/CAM technology, while conventional ARS were fabricated by an experienced dental laboratory technician.

### Randomization and Blinding (See Supplementary Material 1)

#### Random sequence generation

A computer-generated random number sequence was produced using SPSS software (version 26, IBM Corp.) by an independent statistician not involved in patient recruitment or outcome assessment.

#### Stratification

Randomization was stratified by laterality (unilateral vs. bilateral involvement) to ensure balance between groups for this important prognostic factor.

#### Allocation concealment

The allocation sequence was implemented using sequentially numbered, opaque, sealed envelopes (SNOSE). The envelopes were prepared and kept by the trial coordinator. After obtaining written informed consent and confirming eligibility, the treating clinician opened the next envelope in sequence to reveal the group assignment.

#### Blinding

Due to the nature of the interventions, patients and the clinicians delivering the treatment could not be blinded. However, outcome assessors [radiologists evaluating MRI scans for disc recapture, technicians measuring condylar volume from cone-beam computed tomography (CBCT), and researchers administering questionnaires] were blinded to group assignment.

### Interventions

#### D-ARS group

##### Design & fabrication

**Fig. 2:**
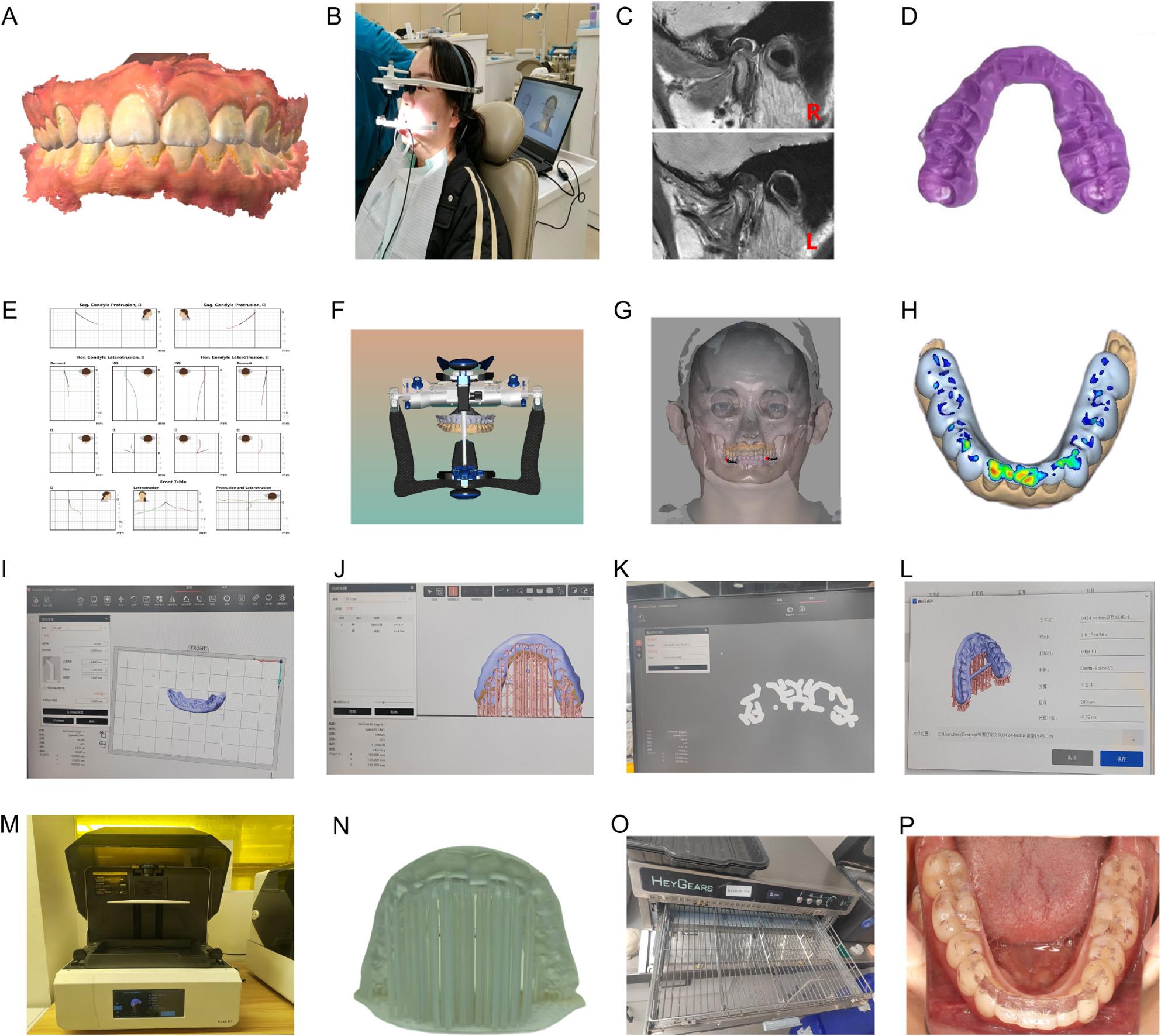
Digital workflow for fabricating the digitally designed anatomical repositioning splint (D-ARS). **A **Intraoral scanning. **B **Electronic axiography for recording condylar movement. **C **Magnetic resonance imaging for assessing disc-condyle relationship. **D **Acquisition of occlusal registration. **E **Individualized mandibular movement data. F Virtual articulator mounting in exocad software. **G **Generation of a dynamic virtual patient through multimodal data registration and fusion. **H **Virtual design of the occlusal surface, showing contact with the opposing dentition. **I **Parameter setting for the digital splint model in Rayshape Panel software. **J **Support structure generation. **K **Slicing of the splint model. **L **Model integrity preview and confirmation before print file generation. **M **3D printing. **N **Printed splint before support removal. **O **Double-sided light curing of the splint in a curing chamber. **P **Intraoral view of the delivered D-ARS in place

Intraoral digital scans (Trios, 3Shape, Fig. 2A), face Scan (3dMD), mandibular motion trajectory (JMAnalyser+, Zebris, Fig. 2B) and CBCT data were merged using exocad software (DentalCad v3.2). The therapeutic mandibular position was first determined clinically at the point of joint click disappearance. The patient was seated in an upright position, and the clinician gently guided the patient's mandible forward and downward. The position was adjusted until the joint click completely disappeared on auscultation (with a stethoscope). This position was then temporarily maintained with a bite wax, and MRI was subsequently taken to confirm successful disc recapture (Fig. 2C). Once confirmed, a definitive occlusal registration was taken using vinyl polysiloxane (VPS) addition‑curing silicone (MeiJiaYin, China, Fig. 2D). Both dental arches were then scanned with an intraoral scanner, and the resulting digital bite registration was matched to the digital maxillary and mandibular models. The matched maxillary and mandibular models (with the bite registration) were then imported into exocad software, together with the occlusal fork and electronic facebow data (Fig. 2E), for virtual articulator mounting (Fig. 2F). Next, the mandible was segmented from the CBCT data and registered into the digital model. The facial scan data were then registered and fused. This process constructed a dynamic virtual patient (Fig. 2G), enabling visualisation of individualised mandibular movement. Based on these movement characteristics, the splint design was adjusted to achieve anatomical posterior cusp adaptation and optimised occlusal contacts, with parameters including wall thickness and extension range defined to generate the final splint model (Fig. 2H).

The splint was fabricated using a 3D printer (Rayshape, China). The digital splint model was imported into Rayshape Panel software to set layer thickness, occlusal surface orientation, internal compensation (Fig. 2I), and support structures (Fig. 2J). After slicing (Fig. 2K) and verifying model integrity, a print file was created (Fig. 2L). The resin was poured into the tank, and printing was initiated (Fig. 2M). Upon completion, the printed splint was removed from the platform (Fig. 2N), detached from supports, ultrasonically cleaned, dried, double-sided light-cured (Fig. 2O), ground, polished, and disinfected. Finally, the splint was delivered intraorally (Fig. 2P).

##### Protocol

As recommended by most previous studies [13], patients were instructed to wear the splint full-time (24 h per day), including during meals, to maintain continuous disc recapture and mandibular advancement. The splint was removed only for tooth brushing and was to be reinserted immediately afterward. Patients were explicitly advised to keep the splint in place during all meals to avoid interruption of disc recapture. Compliance was monitored through patient diaries and follow-up interviews.

#### ARS group

##### Design & fabrication

Conventional alginate impressions were taken and poured in dental stone. The therapeutic mandibular position was determined and the definitive occlusal registration was taken using the same procedure as described for the D-ARS group (Section "D-ARS group"). The VPS addition‑curing silicone occlusal registration was then transferred to a mean-value articulator to fabricate the ARS. A conventional hard acrylic resin anterior repositioning splint was fabricated on the plaster model using a standard laboratory protocol.

##### Protocol

Identical wearing instructions and mandibular positioning as the D-ARS group.

#### Common care

**Fig. 3:**
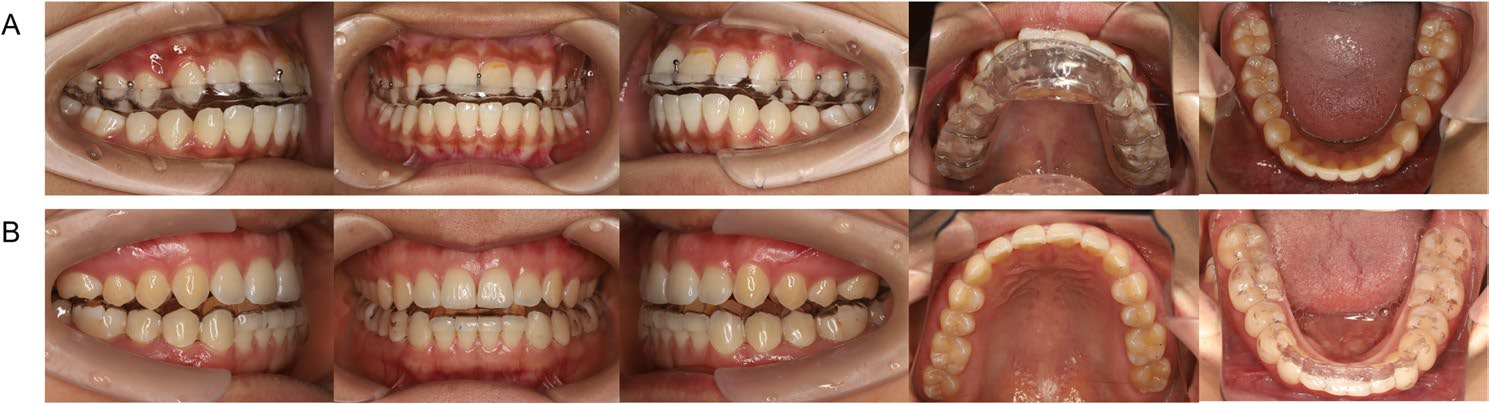
Intraoral views of the two types of occlusal splints. **A** The conventional anterior repositioning splint (ARS). **B** The digitally designed anatomical repositioning splint (D-ARS)

Both groups received identical patient education regarding splint care, dietary recommendations, and the importance of compliance. Follow-up appointments were scheduled at 1 week (for adjustment), 1 month, 3 months, and 6 months. After 6 months of follow-up, outcomes were assessed using MRI, CBCT, and the Oral Impacts on Daily Performances (OIDP) questionnaire. Figure 3 shows intraoral photographs of the two types of occlusal splints in place. No concomitant care or interventions were received by participants in either group during the trial period.

### Outcomes

#### Primary outcome

##### Disc recapture success rate

**Fig. 4:**
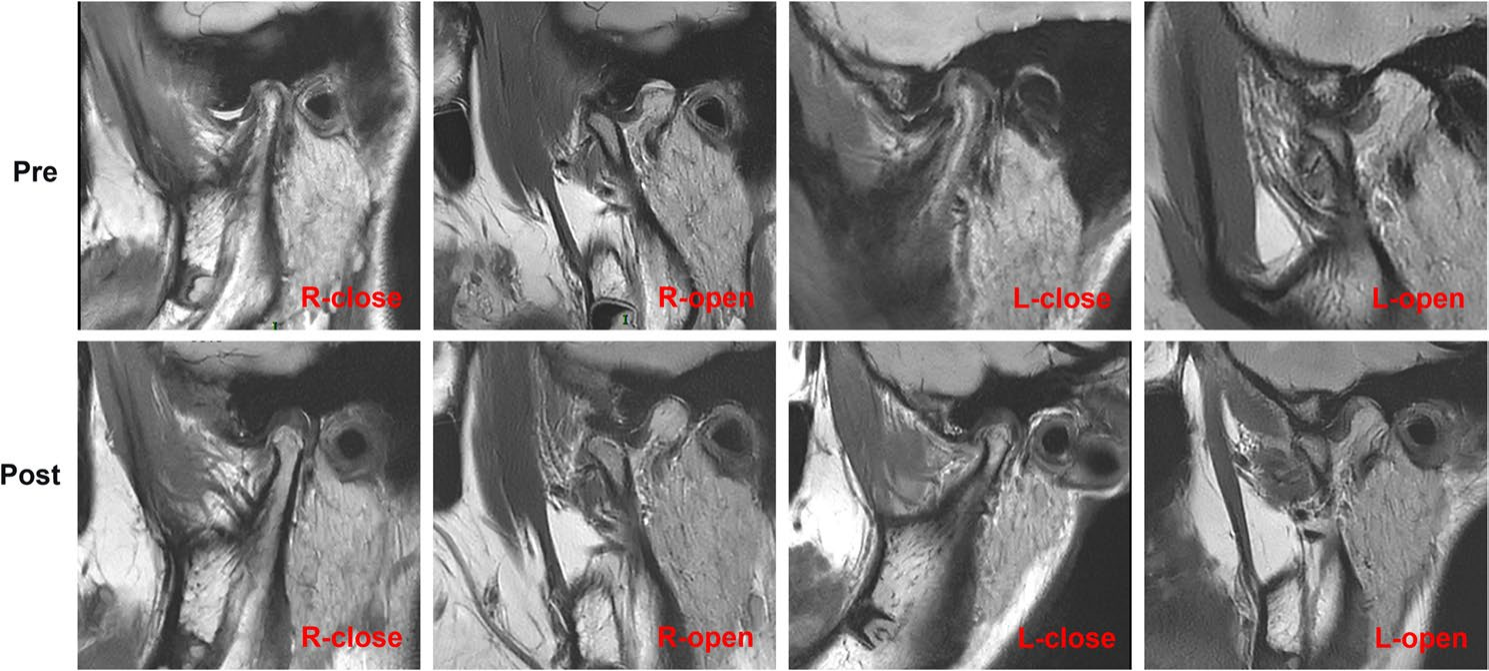
Pre- and post-treatment magnetic resonance imaging (MRI) of a representative patient. Pre: before treatment; Post: after treatment; R-close: right temporomandibular joint (TMJ), closed-mouth position; L-close: left TMJ, closed-mouth position; R-open: right TMJ, open-mouth position; L-open: left TMJ, open-mouth position

Assessed via MRI at the 6-month follow-up. An independent and blinded panel of radiologists reviewed the imaging data to adjudicate whether anatomical disc repositioning was achieved. Success was defined as the restoration of a normal disc-condyle relationship (posterior band of the disc at the 12 o’clock position relative to the condyle) on sagittal MRI slices in closed-mouth position (See Fig. 4).

#### Secondary outcomes

##### Condylar volume change

As per the methodology established in prior literature [14], three-dimensional reconstruction of the condyle was performed using 3D Slicer software (version 5.0.3). The specific steps were as follows: (1) Pre- and post-treatment CBCT data in DICOM format were imported into the software. The images were aligned in the axial, sagittal, and coronal planes. The Frankfort horizontal plane was set parallel to the axial plane, and the midsagittal plane was aligned through the nasal septum and the center of the foramen magnum. Both the highest points of the bilateral glenoid fossae were positioned on the same axial plane. After adjusting the window width and window level, the “CT‑Bone” mode was selected. (2) The “Segment Editor” module was used to reconstruct the maxilla and mandible. The mandible was segmented via the “Grow from seed” tool to generate its three‑dimensional model. The maxilla was reconstructed using the “Threshold” tool (threshold range: 400 to maximum value), followed by removal of the mandibular structure. The segmentation contours of both jaws were inspected and refined layer by layer, resulting in separate 3D models of the maxilla and mandible before and after treatment. (3) On sequentially examined axial slices, the first slice revealing the lowest point of the mandibular sigmoid notch was defined as the inferior boundary of the condyle. Using this anatomical landmark, the registered mandibular model was segmented to isolate a three-dimensional model of the condyle, the volume of which was subsequently recorded.

##### Patient-reported wearing comfort

Evaluated using the OIDP questionnaire, scored as a percentage (0–100%), with lower scores indicating better comfort and fewer oral impacts.

**Measurement timepoints**

All outcomes were measured at baseline (except disc recapture) and at the 6-month follow-up.

### Statistical analysis

#### Analysis principles

The primary analysis followed the intention-to-treat (ITT) principle, including all randomized patients in the groups to which they were assigned. A per-protocol analysis was also performed for sensitivity analysis.

#### Descriptive statistics

Continuous variables with normal distribution were presented as mean ± standard deviation (SD); non-normally distributed data as median (interquartile range, IQR). Categorical variables were presented as frequency (percentage). 

#### Comparative statistics

##### Primary outcome

Between-group comparison of disc recapture rates was performed using the Chi-square test (or Fisher’s exact test if expected cell counts were <5) 

##### Secondary outcomes

1.  (Within-group changes in condylar volume were analyzed using the paired ttest for the D-ARS group and the Wilcoxon signedrank test for the ARS group, based on data distribution. Betweengroup differences in volume change were compared using the MannWhitney U test.
2. OIDP scores were compared between groups using independent t-tests.Normality was assessed using the Shapiro-Wilk test. Homogeneity of variances was assessed using Levene’s test.

Normality was assessed using the Shapiro-Wilk test. Homogeneity of variances was assessed using Levene’s test.

#### Software and significance

All analyses were conducted using SPSS version 26.0 (IBM Corp.). A two-sided P-value < 0.05 was considered statistically significant.

#### Ancillary analyses

No subgroup analyses were performed in this study. The per-protocol analysis, conducted as a sensitivity analysis, was pre-specified in the registered trial protocol (ChiCTR2400080947). All other statistical analyses reported in this manuscript were performed as originally planned; no post-hoc analyses were conducted.

## Results

### Patient flow and baseline characteristics

A total of 100 patients were assessed for eligibility, of whom 18 were excluded (6 did not meet the inclusion criteria and 12 declined to participate). The remaining 82 patients were randomized and allocated to the two study groups based on a stratification scheme: 30 patients with bilateral reducible anterior disc displacement (ADDwR) and 52 with unilateral ADDwR (Fig. 1). Of these, 41 patients were allocated to the digitally designed anatomical repositioning splint (D‑ARS) group and 41 to the conventional anterior repositioning splint (ARS) group. All allocated patients received the intended intervention. During the follow‑up period, 1 patient in the D‑ARS group and 2 patients in the ARS group were lost to follow‑up for personal reasons. Consequently, 40 patients in the D‑ARS group and 39 patients in the ARS group completed the study and were included in the final per‑protocol analysis.

Baseline characteristics of the initially enrolled patients (*n* = 82) are presented in Table 1. The mean age was comparable between the D‑ARS group (13.83 ± 1.21 years) and the ARS group (13.73 ± 1.40 years; t = 0.354, *p* = 0.724). No significant differences were observed in gender distribution (male: 31.71% vs. 34.15%; χ² = 0.055, *p* = 0.814) or in the laterality of ADDwR (bilateral: 36.59% in both groups; χ² = 0.000, *p* = 1.000). Each group contributed 56 joints (bilateral cases counted as two joints) for subsequent joint‑level analyses.

**Table 1:** Baseline characteristics of initially enrolled patients

	D-ARS (*n* = 41)	ARS (*n* = 41)	Statistic	*P*-Value
Age (years, mean ± SD)	13.83 ± 1.21	13.73 ± 1.40	T = 0.354	0.724
Gender [n (%)]			χ² = 0.055	0.814
Male	13 (31.71)	14 (34.15)		
Female	28 (68.29)	27 (65.85)		
ADDwR [n (%)]			χ² = 0.000	1.000
Bilateral	15 (36.59)	15 (36.59)		
Unilateral	26 (63.41)	26 (63.41)		
Total Joints (n)	56	56	—	—

Among the patients who completed the follow‑up (*n* = 79), baseline comparability remained well preserved (Table 2). The mean age was 13.83 ± 1.21 years in the D‑ARS group (*n* = 40) and 13.59 ± 1.33 years in the ARS group (*n* = 39) (t = 0.851, *p* = 0.398). Gender proportion (male: 30.0% vs. 33.3%; χ² = 0.064, *p* = 0.801) and ADDwR laterality (bilateral: 35.0% vs. 38.5%; χ² = 0.060, *p* = 0.807) were also similar between the two groups. A total of 54 joints were evaluated in each group at the final assessment. The balanced baseline profiles support the validity of subsequent between‑group comparisons.

**Table 2:** Baseline characteristics of patients who completed follow-up

	D-ARS (*n* = 40)	ARS (*n* = 39)	Statistic	*P*-Value
Age (years, mean ± SD)	13.83 ± 1.21	13.59 ± 1.33	T = 0.851	0.398
Gender [n (%)]			χ² = 0.064	0.801
Male	12 (30.0)	13 (33.3)		
Female	28 (70.0)	26 (66.7)		
ADDwR [n (%)]			χ² = 0.060	0.807
Bilateral	14 (35.0)	15 (38.5)		
Unilateral	26 (65.0)	24 (61.5)		
Total Joints (n)	54	54	—	—

### Disc recapture success rate

The disc recapture success rates for both groups at the 6‑month follow‑up are summarized in Table 3. In the D-ARS group (54 joints total), disc recapture was unsuccessful in 3 joints and successful in 51 joints. In the ARS group (54 joints total), disc recapture was unsuccessful in 11 joints and successful in 43 joints. In the per-protocol analysis, which included only patients who completed the 6-month follow-up (D-ARS: 54 joints; ARS: 54 joints), the recapture rates were 94.44% and 79.63%, respectively (*p* = 0.022).

**Table 3:** Comparison of disc reposition success rates between the two groups

Group	Total Joints (*n*)	Successful Reposition *n* (%)	Unsuccessful Reposition *n* (%)
D-ARS	54	51 (94.44)	3 (5.56)
ARS	54	43 (79.63)	11 (20.37)
*P-Value*	0.022		

A chi-square test was used to compare the success rates between the two groups, with the significance level set at α = 0.05

Under the primary ITT analysis with worst-case imputation for the 4 missing joints (2 in D-ARS, 2 in ARS), the disc recapture rate was 94.4% (51/54) in the D-ARS group and 83.3% (45/54) in the ARS group (*p* = 0.022). The results and statistical significance were consistent between the primary ITT analysis and the per-protocol sensitivity analysis, indicating that the findings were robust to different methods of handling missing data.

### Condylar volume change

**Fig. 5:**
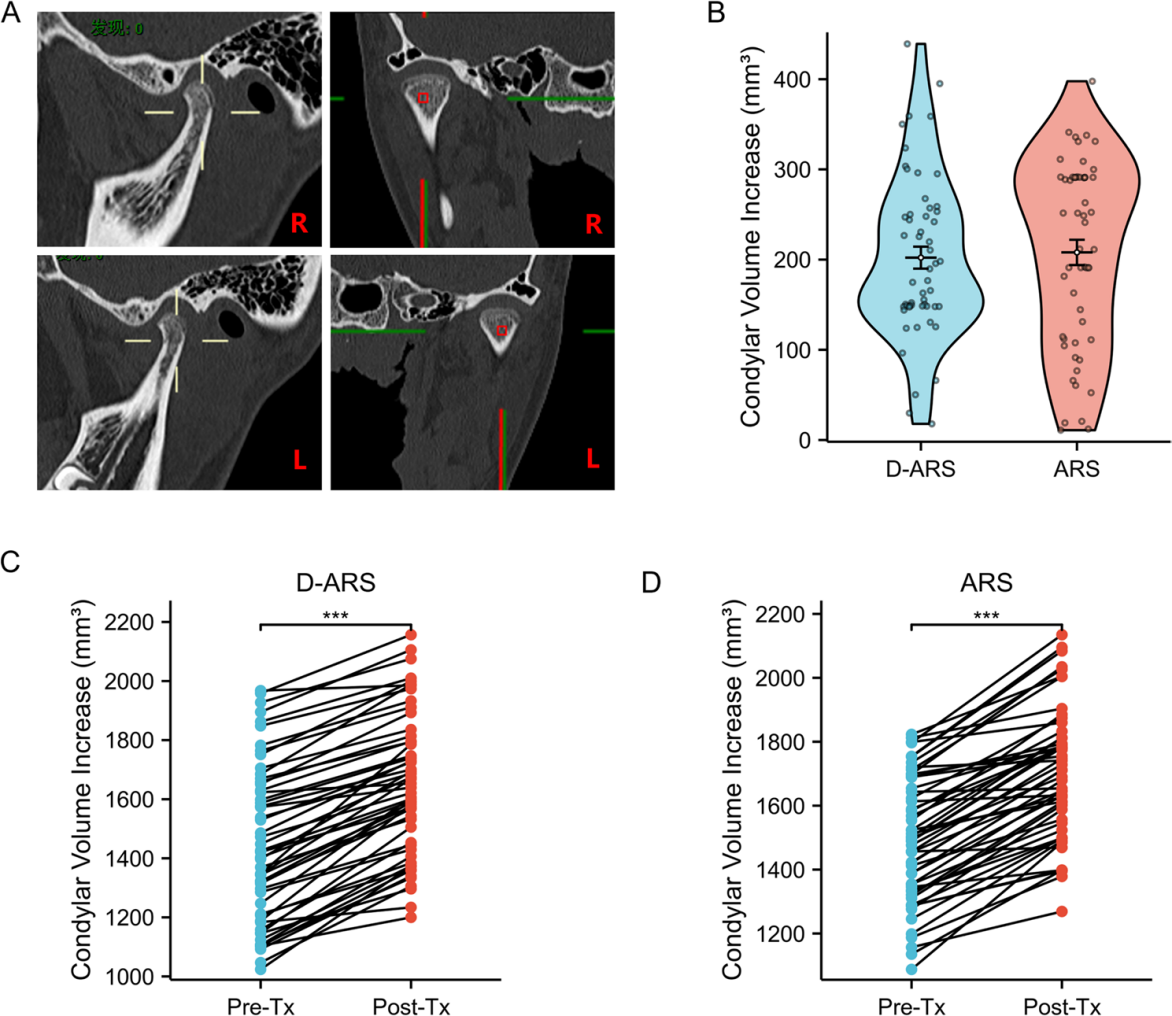
Condylar changes before and after treatment. D‑ARS: digitally designed anatomical repositioning splint group; ARS: conventional anterior repositioning splint group. **A** Representative post‑treatment cone‑beam computed tomography (CBCT) image showing the condyle. **B** Violin plot comparison of condylar volume change (pre‑treatment vs. post‑treatment) between the D‑ARS and ARS groups. Both groups showed a significant increase after treatment, with no statistically significant difference between the two groups (Mann‑Whitney U test). **C** Paired comparison plot for the D‑ARS group: each line connects the pre‑treatment and post‑treatment volume of a single condyle. Most lines slope upward, indicating consistent growth. **D** Paired comparison plot for the ARS group, presented in the same format. A general increase is also observed, with slightly higher inter‑condyle variability

As shown in Fig. 5A, post‑treatment CBCT revealed notable new bone formation on the condylar surface. Figure 5C and D present paired individual data: each grey line connects baseline and post‑treatment volume of one condyle. In the D‑ARS group (Fig. 5C), most lines slope upward, confirming a consistent volume increase at the individual joint level. The ARS group (Fig. 5D) also shows a general upward trend, though with slightly greater inter‑condyle variability. Figure 5B compares the condylar volume changes between the two groups, and the detailed statistics are provided in Supplementary Material 2. In the D‑ARS group, the mean condylar volume increased from 1498.3 ± 287.5 mm³ at baseline to 1702.1 ± 275.8 mm³ post‑treatment, with a mean volume gain of 203.8 mm³ (95% CI: 177.7–229.9; *p* < 0.001, paired t‑test). In the ARS group, condylar volume increased from 1492.6 ± 213.4 mm³ to 1701.5 ± 248.9 mm³, corresponding to a mean increase of 208.9 mm³ (95% CI: 178.8–239.0; *p* < 0.001, Wilcoxon signed‑rank test). The Mann‑Whitney U test revealed no statistically significant difference between the two groups (*p* = 0.5615). These results indicate that both splint modalities effectively promoted condylar growth, with no significant difference in condylar volume increase between the D-ARS and ARS approaches.

### Patient-reported wearing comfort

**Fig. 6:**
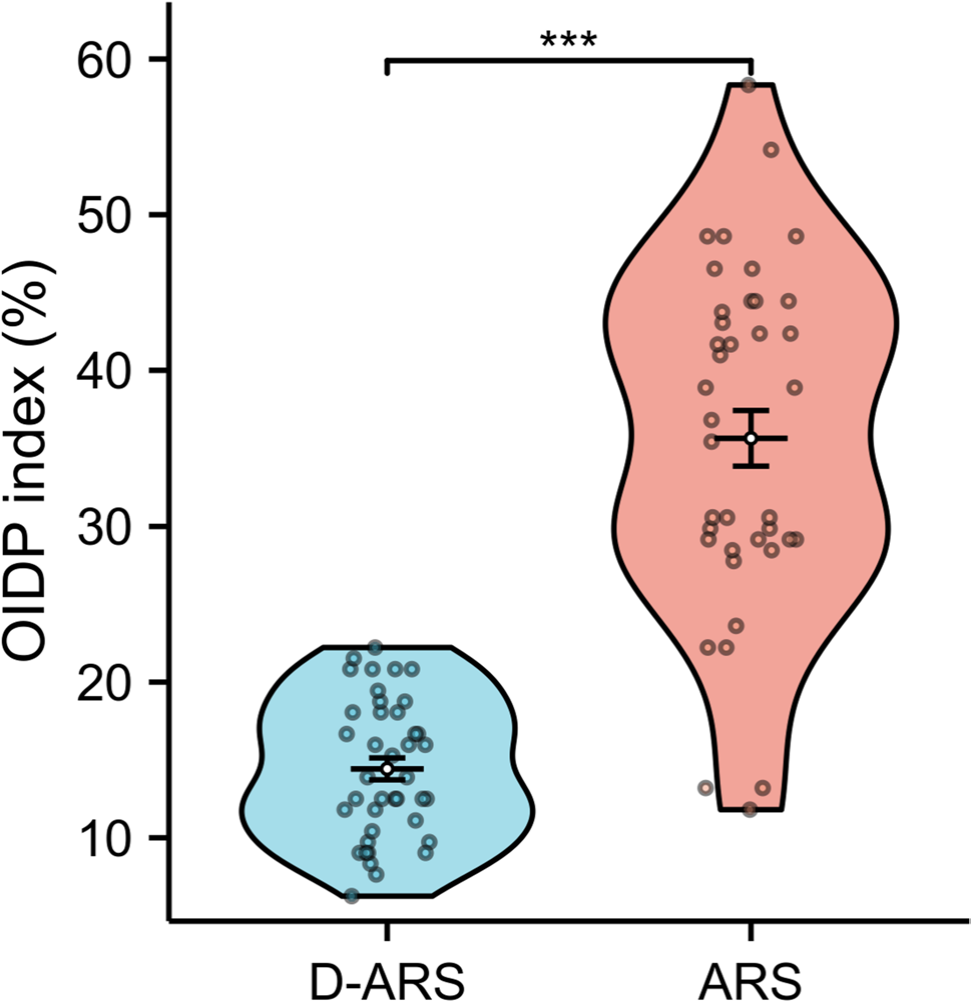
Comparison of OIDP scores between the two treatment groups. D-ARS, digitally designed anatomical repositioning splint group; ARS, conventional anterior repositioning splint group

No serious adverse events were reported in either group during the 6-month follow-up period. No patient discontinued treatment due to adverse effects. Patient-reported wearing comfort, assessed using the OIDP scale, showed a significant improvement in the D-ARS group compared to the conventional ARS group after 6 months of treatment. The median OIDP score in the D-ARS group was 14.4% (interquartile range [IQR]: 9.0–20.8), significantly lower than that in the ARS group, which was 35.6% (IQR: 29.2–44.4) (*p* < 0.001, Mann-Whitney *U* test). The distribution of OIDP scores for the two groups is visually summarized in Fig. 6 and Supplementary Material 3. These results indicate that patients treated with the digitally designed anatomical repositioning splint experienced substantially fewer oral-health-related impacts on daily performance, reflecting superior wearing comfort and better overall treatment experience compared to those treated with the conventional splint.

## Discussion

This randomized controlled trial investigated the clinical efficacy of D‑ARS versus conventional ARS for treating ADDwR adolescents. The primary finding was that D‑ARS achieved a significantly higher rate of disc recapture and superior patient‑reported comfort compared to ARS after six months of treatment, while both modalities were equally effective in promoting condylar growth. Consequently, the null hypothesis—postulating no difference in clinical performance between the two splints—is partially rejected.

Incorporating findings from prior literature, persistent intra-articular derangements such as disc displacement may function as adverse biomechanical stimuli that disrupt adaptive condylar growth, potentially leading to mandibular dysplasia and facial asymmetry [15]. Even in young patients, temporomandibular joint disc displacement has been closely associated with early degenerative joint disease, characterized by progressive structural deterioration [16]. For adolescents in active growth phases, proactive early intervention—such as attempts to recapture the displaced disc—is therefore warranted to restore the normal disc-condyle relationship and thereby mitigate potential inhibitory effects on mandibular development [17]. As a non-invasive, reversible, and cost-effective treatment modality, functional splint therapy represents a key conservative approach in the management of disc displacement, with ARS being a well-established mainstream option for reducible anterior disc displacement [13].

The success rate of disc recapture with ARS is directly influenced by the reducibility of the disc displacement and patient compliance with splint wear [5]. Prior to treatment initiation, the target disc position was confirmed via MRI, and the ARS was designed and fabricated accordingly. Patients were instructed to wear the splint full‑time (24 h per day). The criteria for determining successful disc recapture in this study were informed by limitations noted in earlier research [18]. Previous evaluations of ARS outcomes have sometimes relied solely on the disappearance of clicking during clinical examination [19] or classified any improvement in disc‑condyle relationship as treatment success, which may overestimate the actual success rate. To enhance reliability, the present study applied a stricter morphological criterion: “a normal disc‑condyle relationship (posterior band of the disc at the 12 o’clock position relative to the condyle) on sagittal MRI slices in the closed‑mouth position.” All MRI assessments were adjudicated by an independent, blinded radiological review panel, which further strengthens the validity of the reported outcomes.

The results of this study demonstrated that the success rate of disc recapture was 94.4% in the D-ARS group compared with 79.6% in the conventional ARS group in the per protocol analysis, and this difference remained statistically significant even under the conservative ITT analysis using worst-case imputation for missing data. The consistency between ITT and per-protocol analyses underscores the robustness of the findings. Clinical studies have confirmed that ARS is generally more effective than other splint designs for managing ADDwR [19]. Both the conventional ARS and the D-ARS function by stabilizing the mandible in an anteriorly repositioned state, thereby contributing to improved disc recapture rates. However, the two splints differ substantially in their design and fabrication, which likely explains the superior patient adherence and higher disc recapture success observed with the D-ARS. Beyond the fabrication workflow, the D-ARS and conventional ARS differ in several key design aspects. First, regarding posterior occlusal support, the D-ARS incorporates anatomically contoured posterior cusps based on the patient’s individual mandibular movement data, providing stable and physiological occlusal contacts.

In contrast, the conventional ARS typically has a flat posterior platform. Second, the anterior ramp design differs substantially: the conventional ARS is fabricated for the maxillary arch and includes a prominent anterior ramp on the lingual aspect of the upper anterior teeth [20], whereas the D-ARS is designed for the mandibular arch with a guidance slope. Because its anatomical posterior occlusal surface stabilises the mandibular position, D-ARS does not require such a ramp, thereby improving patient comfort and speech. Third, regarding material and accuracy, the D-ARS is 3D-printed from a biocompatible resin with high precision, while the conventional ARS is hand-moulded from heat-cured acrylic resin. These design differences likely contribute to the superior patient-reported comfort and higher adherence observed with the D-.

RS, which in turn may promote more stable disc recapture. The reported success rates of conventional ARS therapy for disc recapture exhibit considerable heterogeneity in the literature, with estimates ranging from 40% to 90%. This heterogeneity likely stems from several factors, including differences in patient demographics (e.g., age), the specific clinical or imaging criteria used to define “success,” and variations in follow-up duration across studies [21]. Furthermore, treatment response is not uniform. While some patients achieve complete restoration of the disc-condyle relationship, others exhibit only limited improvement or no response at all. This variability may be influenced by factors such as the chronicity of the condition, the degree of initial disc displacement, and patient compliance with splint wear [5]. The role of the retrodiscal tissue (RDT) has also been highlighted as potentially crucial in this biomechanical process [22]. A dense and well-developed RDT is considered important for stabilizing the disc and facilitating coordinated condylar movement [23]. Nonetheless, the precise underlying mechanisms determining therapeutic success remain incompletely understood and warrant further investigation.

Both groups exhibited an increase in condylar volume, which aligns with previous literature reporting that ARS can facilitate regenerative condylar remodeling [19]. Notably, the characteristic “double-contour” sign on imaging—an indicator of active condylar surface remodeling—was observed in approximately 80% of patients following ARS therapy [24, 25]. In contrast to ADDwR, ADDwoR significantly increases the loading on the condylar surface, which over time can lead to cartilage degradation and subchondral bone destruction [26]. By comparison, repositioning the disc–condyle relationship can effectively reduce this mechanical burden. ARS therapy functions by pulling the condyle forward and downward, which helps to maintain the restored disc–condyle relationship, decreases articular pressure, and may enhance biomechanical stimulation, improve blood flow, and thereby promote osseous repair and regeneration [27]. Additionally, all participants in this study were adolescents (12–16 years old), suggesting that a portion of the observed volume increase may be attributable to ongoing physiological condylar growth. The difference in disc recapture success between groups was not accompanied by a statistically significant difference in the magnitude of condylar growth. This suggests that ADDwR may have a relatively minor inhibitory effect on condylar volume—unlike its irreversible counterpart (ADDwoR), which is often associated with more pronounced growth restraint [14]. These observations align with the findings of Wang et al. [28], who reported that mandibular advancement effectively promotes condylar remodeling in adolescent patients with Class II malocclusion, regardless of the presence of disc displacement, while also noting that condylar growth in ADDwR joints remained comparable to that in normal joints, whereas growth in ADDwoR joints was significantly reduced. Finally, the 6‑month follow‑up period may be insufficient to detect small differences in condylar volume change. However, studies indicate that unintervened ADDwR carries a clear risk of progression to ADDwoR [29]. As anterior disc displacement advances, the posterior band of the disc often becomes enlarged, may fold or perforate, and is frequently accompanied by synovitis along with osteoarthritic changes such as subchondral bone resorption and destruction of the condylar surface [30]. In follow-up exceeding two years, condylar bone deterioration was observed to worsen in 17% of adolescents diagnosed with temporomandibular joint degenerative disease [31]. These findings underscore the clinical importance of early and active intervention in ADDwR.

Nevertheless, the D-ARS group reported markedly better wearing comfort, as reflected in significantly lower OIDP scores. The bulky anterior ramp and lack of posterior support in conventional ARS often impair masticatory function and speech, reducing patient adherence. In contrast, the anatomically contoured posterior cusps and reduced anterior profile of the D-ARS likely distribute occlusal forces more physiologically, improving comfort and compliance—a critical factor in long-term treatment success.

The integration of digital design and fabrication represents a paradigm shift in splint therapy. By utilizing CBCT-derived anatomical data and multimodal registration, the D-ARS can be customized to each patient’s condylar trajectory and dental morphology, enabling more precise and stable mandibular positioning. This digital workflow not only enhances therapeutic accuracy but also streamlines the fabrication process, reducing laboratory time and potential manual errors.

Several limitations of this study should be acknowledged. First, the follow‑up period was limited to six months; longer‑term investigations are required to evaluate the stability of disc recapture and condylar remodeling. Existing evidence suggests that while ARS therapy demonstrates favorable short‑term outcomes in disc repositioning, its long‑term efficacy may be less consistent [11]. Further studies are therefore warranted to assess whether the restored disc‑condyle relationship can be maintained following splint removal. Nevertheless, the 6‑month results demonstrate promising short‑term stability, which provides a foundation for future long‑term investigations. Second, although the sample size was adequate for detecting differences in primary outcomes, it may not be sufficient for detailed subgroup analyses, such as comparisons between unilateral and bilateral involvement. Due to sample size constraints, this study did not employ a mixed‑effects model, which would otherwise be the appropriate method to account for within‑subject correlation in bilateral joint measurements. Future studies should incorporate such analytical methods for validation. Third, the present study was conducted exclusively in adolescents with ADDwR. Nevertheless, considering that condylar growth continues until approximately 21–22 years of age [25], young adults with ADDwR may also benefit from active intervention aimed at reestablishing normal disc‑condyle relationships to support ongoing mandibular development and mitigate the risk of progressive degenerative changes.

## Conclusions

In adolescents with ADDwR, a D-ARS achieved a higher rate of disc recapture and better patient comfort compared to a conventional ARS after 6 months of treatment, while both were equally effective in promoting condylar growth. The integration of digital design and manufacturing offers a promising, patient-centered approach to splint therapy, with the potential to improve clinical outcomes and adherence. longer‑term follow‑up studies are warranted to evaluate the durability of disc recapture and the sustainability of condylar adaptive changes.

## Supplementary Information

Supplementary Material 1. Stratified Randomization Schedule for the Trial (D-ARS vs ARS)

Supplementary Material 2: Table S1. Condylar Volume Changes Following Treatment with D-ARS and ARS

Supplementary Material 3: Table S2. Comparison of Patient Reported Wearing Comfort Between Treatment Groups Using OIDP Scores

Supplementary Material 4. CONSORT 2025 checklist of information to include when reporting a randomised trial

## Data Availability

The datasets generated and/or analysed during the current study are not publicly available due to privacy concerns but are available from the corresponding author on reasonable request.

## Abbreviations

ADDwR: Anterior disc displacement with reduction
TMJ: Temporomandibular joint
ARS: Anterior repositioning splints
D-ARS: Digitally designed anatomical repositioning splint
MRI: Magnetic resonance imaging
CBCT: Magnetic resonance imaging
OIDP: Oral impacts on daily performance
CI: Confidence interval
