# Supplementary material for: Efficacy of a digitally designed anatomical repositioning splint for adolescent temporomandibular joint anterior disc displacement with reduction: a randomized controlled trial
Source: BMC Oral Health. 2026 Jun 5;26:1410. doi: 10.1186/s12903-026-08753-1 (PMC13459629; doi:10.1186/s12903-026-08753-1)
Supplement: Supplementary file 1 [file 12903_2026_8753_MOESM1_ESM.docx]

**Total patients**: 82 (ID 1–82)

**Bilateral cases**: 30 patients (IDs 1–30)

**Unilateral cases**: 52 patients (IDs 31–82)

**Block size**: 4 (ensures balance every 4 patients)

**Allocation ratio**: 1:1 (Group A = D-ARS, Group B = ARS)

**Method**: Computer-generated random sequences within each stratum.

### ****Bilateral Stratum (30 patients)****

- Complete blocks: 30 ÷ 4 = 7 blocks (28 patients) + 1 incomplete block (2 patients).
- Random permutations of "AABB" within each block.

**Randomized bilateral list (IDs 1–30):**

| ID | Stratum | Group |
| --- | --- | --- |
| 1 | Bilateral | A |
| 2 | Bilateral | B |
| 3 | Bilateral | B |
| 4 | Bilateral | A |
| 5 | Bilateral | B |
| 6 | Bilateral | A |
| 7 | Bilateral | A |
| 8 | Bilateral | B |
| 9 | Bilateral | A |
| 10 | Bilateral | B |
| 11 | Bilateral | B |
| 12 | Bilateral | A |
| 13 | Bilateral | B |
| 14 | Bilateral | A |
| 15 | Bilateral | A |
| 16 | Bilateral | B |
| 17 | Bilateral | A |
| 18 | Bilateral | B |
| 19 | Bilateral | B |
| 20 | Bilateral | A |
| 21 | Bilateral | B |
| 22 | Bilateral | A |
| 23 | Bilateral | A |
| 24 | Bilateral | B |
| 25 | Bilateral | A |
| 26 | Bilateral | B |
| 27 | Bilateral | A |
| 28 | Bilateral | B |
| 29 | Bilateral | A |
| 30 | Bilateral | B |

### ****Unilateral Stratum (52 patients)****

- Complete blocks: 52 ÷ 4 = 13 blocks (52 patients exactly).
- Random permutations of "AABB" within each block.

**Randomized unilateral list (IDs 31–82):**

| ID | Stratum | Group |
| --- | --- | --- |
| 31 | Unilateral | B |
| 32 | Unilateral | A |
| 33 | Unilateral | B |
| 34 | Unilateral | A |
| 35 | Unilateral | A |
| 36 | Unilateral | B |
| 37 | Unilateral | B |
| 38 | Unilateral | A |
| 39 | Unilateral | A |
| 40 | Unilateral | B |
| 41 | Unilateral | B |
| 42 | Unilateral | A |
| 43 | Unilateral | A |
| 44 | Unilateral | B |
| 45 | Unilateral | A |
| 46 | Unilateral | B |
| 47 | Unilateral | B |
| 48 | Unilateral | A |
| 49 | Unilateral | B |
| 50 | Unilateral | A |
| 51 | Unilateral | A |
| 52 | Unilateral | B |
| 53 | Unilateral | A |
| 54 | Unilateral | B |
| 55 | Unilateral | B |
| 56 | Unilateral | A |
| 57 | Unilateral | A |
| 58 | Unilateral | B |
| 59 | Unilateral | B |
| 60 | Unilateral | A |
| 61 | Unilateral | A |
| 62 | Unilateral | B |
| 63 | Unilateral | B |
| 64 | Unilateral | A |
| 65 | Unilateral | A |
| 66 | Unilateral | B |
| 67 | Unilateral | B |
| 68 | Unilateral | A |
| 69 | Unilateral | A |
| 70 | Unilateral | B |
| 71 | Unilateral | B |
| 72 | Unilateral | A |
| 73 | Unilateral | A |
| 74 | Unilateral | B |
| 75 | Unilateral | A |
| 76 | Unilateral | B |
| 77 | Unilateral | B |
| 78 | Unilateral | A |
| 79 | Unilateral | B |
| 80 | Unilateral | A |
| 81 | Unilateral | A |
| 82 | Unilateral | B |
