# Supplementary material for: Efficacy of a digitally designed anatomical repositioning splint for adolescent temporomandibular joint anterior disc displacement with reduction: a randomized controlled trial
Source: BMC Oral Health. 2026 Jun 5;26:1410. doi: 10.1186/s12903-026-08753-1 (PMC13459629; doi:10.1186/s12903-026-08753-1)
Supplement: Supplementary file 2 [file 12903_2026_8753_MOESM2_ESM.docx]

**Table S1.** **Condylar Volume Changes Following Treatment with D-ARS and ARS**

| **Parameter** | **D-ARS Group (n = 54 joints)** | **ARS Group (n = 54 joints)** | ***p*-Value** |
| --- | --- | --- | --- |
| **Pre‑treatment volume (mm³)** | 1498.3 ± 287.5 | 1492.6 ± 213.4 | 0.901† |
| **Post‑treatment volume (mm³)** | 1702.1 ± 275.8 | 1701.5 ± 248.9 | 0.989† |
| **Volume change (mm³), mean (95% CI)** | 203.8 (177.7–229.9) | 208.9 (178.8–239.0) | 0.561‡ |
| ***p*-Value (within‑group)** | < 0.001* | < 0.001* | – |

*Note.*Data are presented as mean ± standard deviation unless otherwise indicated. Within‑group comparisons: D-ARS group analyzed with paired t-test; ARS group analyzed with Wilcoxon signed‑rank test (non‑normal distribution of change values). Abbreviations: D‑ARS, digitally designed anatomical repositioning splint; ARS, conventional anterior repositioning splint; † Independent samples t-test; ‡ Mann–Whitney *U* test for between‑group comparison of volume change.
