# Supplementary material for: Efficacy of a digitally designed anatomical repositioning splint for adolescent temporomandibular joint anterior disc displacement with reduction: a randomized controlled trial
Source: BMC Oral Health. 2026 Jun 5;26:1410. doi: 10.1186/s12903-026-08753-1 (PMC13459629; doi:10.1186/s12903-026-08753-1)
Supplement: Supplementary file 3 [file 12903_2026_8753_MOESM3_ESM.docx]

**Table S2. Comparison of Patient‑Reported Wearing Comfort Between Treatment Groups Using OIDP Scores**

| Parameter | D‑ARS Group (n = 40) | ARS Group  (n = 39) | Statistical Analysis |
| --- | --- | --- | --- |
| Mean ± SD (%) | 14.43 ± 4.46 | 35.63 ± 11.15 | Welch t‑test:  t(77) = 11.75,  p*< 0.001* |
| Median (IQR) (%) | 14.4  (9.0–20.8) | 35.6  (29.2–44.4) |  |
| Range (%) | 6.3–22.2 | 11.8–58.3 |  |

*Note.* Data were normally distributed in both groups (Shapiro‑Wilk p > 0.05). Homogeneity of variance was violated (Levene’s test: p < 0.05); therefore, the Welch-t test was used for between group comparison. Abbreviations: D‑ARS, digitally designed anatomical repositioning splint; ARS, conventional anterior repositioning splint; SD, standard deviation; IQR, interquartile range.
